# Building a communication and support network among quality improvement teams in nursing homes: a longitudinal study of the SCOPE trial

**DOI:** 10.1186/s43058-024-00559-y

**Published:** 2024-03-04

**Authors:** Reza Yousefi Nooraie, Qiuyuan Qin, Adrian Wagg, Whitney Berta, Carole Estabrooks

**Affiliations:** 1 Department of Public Health Sciences, University of Rochester, Rochester, NY USA; 2 Department of Medicine, Faculty of Medicine & Dentistry, University of Alberta, Edmonton, AB Canada; 3 Institute of Health Policy, Management, and Evaluation, University of Toronto, Toronto, ON Canada; 4 Faculty of Nursing, University of Alberta, Edmonton, AB Canada

**Keywords:** Social network analysis, Quality improvement, Implementation, Nursing home, Long-term care facilities

## Abstract

**Background:**

We applied a longitudinal network analysis approach to assess the formation of knowledge sharing and collaboration networks among care aide-led quality improvement (QI) teams in Canadian nursing homes participating in the Safer Care for Older Persons (in residential) Environments (SCOPE) trial which aimed to support unregulated front-line staff to lead unit-based quality improvement (QI) teams in nursing homes. We hypothesized that SCOPE's communicative and participatory nature would provide opportunities for peer support, knowledge sharing, and collaboration building among teams.

**Methods:**

Fourteen QI teams in Alberta (AB) and seventeen QI teams in British Columbia (BC) participated in the study. Communications across nursing homes occurred through a series of 4 collaborative Learning Congresses (training sessions) over a 1-year period. The senior leaders of QI teams participated in two online network surveys about the communication/collaboration between teams in their province, 1 month after the first, and 6 months later, after the fourth Learning Congress. We developed communication and collaboration network maps pertaining to three time points: before SCOPE, at 2 months, and at 9 months.

**Results:**

Over time, teams made significantly more new connections and strengthened existing ones, within and across regions. Geographic proximity and co-membership in organizational chains were important predictors of connectivity before and during SCOPE. Teams whose members were well connected at baseline disproportionately improved connectivity over time. On the other hand, teams that did not have prior opportunities to connect appeared to use SCOPE to build new ties.

**Conclusions:**

Our findings suggest the importance of network-altering activities to the formation of collaboration networks among QI teams across nursing homes. Active strategies could be used to better connect less connected teams and facilitate collaboration among geographically proximate teams. These findings may inform the development of interventions to leverage existing networks and provide new networking opportunities to develop and sustain organizational improvements.

**Supplementary Information:**

The online version contains supplementary material available at 10.1186/s43058-024-00559-y.

Contributions to the literature

- Complex team-based interventions are effective in connecting quality improvement teams across health institutions.
- Network formation among teams naturally happens selectively and unequally. Already well-connected teams and teams that are geographically and organizationally closer are more likely to connect over time.
- Intentional network-altering interventions are needed to enrich more equal information exchange and support across teams, especially for rural and less connected nursing homes.

## Introduction

Social networks provide their members with access to knowledge and support and serve as conduits for the dissemination of innovations [1]. Communication networks are particularly important in the context of organizational change and process improvement [2], as individuals’ adoption and implementation of new behavior is influenced by local norms, peer support and pressure, and alignment of local opinion leaders [3]. At the organizational level, learning from the challenges and innovations of other similar organizations and the formation of informal or formal mentorships and inter-organizational partnership affect the implementation and scale up of innovative programmatic innovations [4, 5].

Network-altering interventions are designed to restructure existing networks by facilitating and strengthening connections among network actors, by building bridges to connect separated groups or by changing the position of some actors in the network [6–8]. Interventions that facilitate information-sharing, peer support, and community formation may serve to change social network structures, even though network change was not the original intent of the intervention [1, 8]. Some examples of (incidental) network-altering interventions are group training interventions [9, 10] and environmental facilitation of formal and informal communication among health care professionals [11–13].

Even though it might not be the main intent, interventions that improve communication and collaboration between healthcare staff may facilitates information exchange and teamwork within a care home, which can lead to better quality of care and resident outcomes [14, 15]. In inter-organizational networks, improvement in connectivity across organizations could facilitate formation of “communities of practice,” in which members collaborate to address a common problem [16]. Information sharing and peer support in communities of practice can help healthcare providers break down professional, geographical, and organizational barriers, learn from each other about common challenges and solutions to address them, develop collective expertise, and gain a sense of identity and belonging [9], which results in value creation and efficiency [17]. The need to be connected to networks of support, peer education, and tele-mentoring has been demonstrated in various settings, including among healthcare professionals in rural institutions [18, 19].

Few studies have assessed the development of communities of practice in nursing homes, but some limited evidence supports their effectiveness in facilitating the development of a culture of collaboration and support among nursing home staff, building capacity for evidence-based practice [20] and sustainable quality improvement [21]. Studying the dynamics of network formation in the context of inter-organizational interventions can provide insights onto the social processes that may affect the formation and sustainment of communities of practice.

There is little empirical evidence on the characteristics and success determinants of organizational and inter-organizational network-altering interventions [6]. Natural experiments can provide insight into these complexities. Understanding the structure and dynamics of collaboration networks will inform quality improvement and culturally and contextually sensitive network-altering interventions [22]. However, little is known about the role of network characteristics on partnership development dynamics, and ways to transform these insights into designing capacity-building interventions, although we do know that those effects are multidimensional and context dependent [23, 24].

Social network analysis (SNA) is a powerful approach that provides a theoretical and analytical perspective to understand social relations and inform and evaluate network interventions. Network analysis can provide insight about the patterns of connectivity in social networks, the position and roles of influential actors, smaller embedded clusters, and the relationship between network characteristics and personal and contextual factors [25]. SNA has been used in before-after studies to gauge the influence of interventions intended to perturb or change existing social networks, or to create stronger ties among previously weakly linked network members. SNA can help advance our understanding of how the implementation of systemic interventions can influence the dynamics of connectivity and formation of communities of support and influence and inform efforts to facilitate the implementation of organizational innovations [25, 26].

In this study, we applied a network analysis lens to assess the formation of knowledge sharing and collaboration networks among participants in the Safer Care for Older Persons (in residential) Environments (SCOPE) intervention, which aims to develop QI skillsets among care staff in nursing homes. SCOPE (NCT03426072) was a pragmatic 12-month clinical trial of a multi-component intervention designed to equip care aides in Canadian nursing homes (sometimes termed as personal support workers or nursing assistants) with skills to lead, with facilitated support, a QI initiative in their nursing home [27–29]. The QI initiative intended to improve quality of care for residents and, at the same time, increase the levels of work engagement and job satisfaction for care aides [27]. While SCOPE was not designed as a network-altering intervention, it has many components aligned with network-altering strategies, as was laid out in the typology of network alteration interventions [6], including group formation, providing motivations to members to connect and build relationships, providing spatial insights to network actors about other teams to collaborate with, and an infra-structure and environment that facilitates connectivity and collaboration across teams. Its communication-based nature could potentially alter the networks among participants, and in doing so, it might facilitate the acquisition of QI skills through information exchange and providing better access to resources.

In accordance with the STROBE checklist of items included in reports of observational studies (Additional file 1), we report on the impact of a communication-based QI intervention, with several opportunities for peer support and feedback, on the formation of communication and collaboration networks across nursing homes.

## Methods

We assessed the effects of the SCOPE intervention on the formation and strengthening of social networks between participating QI teams. Given the inherently collaborative nature of the intervention, we hypothesized that being involved in the SCOPE trial would improve information exchange and support networks.

### Context

Fourteen nursing home QI teams in Alberta (AB) and seventeen in British Columbia (BC) participated in the study. The QI teams were led by care aides, who are unregulated workers in nursing homes similar to nursing assistants in US and personal support workers in other jurisdictions. The teams usually involved unit managers and facility managers as “team sponsors” and “senior sponsors” respectively.

SCOPE provided training for QI skills identified in the Institute for Health Improvement’s (IHI) *collaborative breakthrough ser*ies model [30] (e.g., collaborative processes of Plan-Do-Study-Act and emphasizing pre-post measurement). While SCOPE focused on equipping care aide teams and senior sponsors to conduct care aide led QI initiatives at the unit or clinical microsystem level, several aspects of the intervention provided opportunities for QI teams to interact. Communications across nursing homes occurred through a series of 4 sessions called Learning Congresses, completed over a 1-year implementation period, led by experienced quality advisors, through which QI skills were introduced and practiced and several opportunities for education, coaching, team building, communication, and networking among participating teams were provided. The Learning Congresses consisted of short plenary presentations on the QI models, group exercises on measuring quality, team building, and sessions focusing on the progress of each participating team. Participating teams had the opportunity to communicate with other teams about their challenges and experiences. Quality Improvement advisors provided continued support to the teams in between Learning Congresses. More details of the SCOPE are provided elsewhere [27–29].

### Recruitment

The sponsors (most often a unit manager) and senior sponsors (most often a director of care from the facility) of SCOPE QI teams were invited to participate in a series of self-administered online surveys about the communication/collaboration between their team and other SCOPE teams in their region and provinces. In Alberta, these were divided into the Edmonton zone, Central and Calgary zones of Alberta Health Services and in British Columbia (BC) the Fraser and Interior Regional Health authorities. The invitational email explained the objectives of the network study, expectations and responsibilities of participants, and efforts to protect confidentiality and privacy. The study was approved by the Research Ethics Boards at the University of Alberta (Pro00082213) University of British Columbia, Fraser Health and Interior Health, and University of Toronto.

### Network survey

Each QI team sponsor/senior sponsor responded to an online survey through which they identified their team’s level of communication and collaboration with all other teams participating in their province. The survey included a scale modified from Harris et al. [31] which contained the following options to the question “Please check the item that best describes your SCOPE team’s current relationship with other SCOPE teams located in your region. Please answer on behalf of your team”:

- *No inter-team relationships*: To date, we have developed little or no relationships with this team
- *Knowing each other*: We have made connections with members of this team about who they are, what they are working on, and how we can help one another
- *Communication at the sponsor level and/or team members*: We share useful information and ideas with the members of this team when opportunity arises
- *Collaboration*: We work together formally or informally to achieve common goals

The survey (Additional file 2) was administered at two time points: 1 month after the SCOPE QI teams participated in the first Learning Congress (November 2018) and 6 months later after the fourth Learning Congress (May 2019). All Learning Congresses were held regionally.

The survey administered after the first Learning Congress also included a yes/no question about whether they had any relationship prior to the start of the SCOPE study. Teams that only participated in second round (after the fourth Learning Congress) also answered questions about relationships before participation in the SCOPE. This way, we captured the information about relationships prior to the start of the SCOPE study purposively at both rounds of data collection.

In other words, we collected three rounds of communication and collaboration data (prior to SCOPE, after Learning Congress 1 (first follow-up) and after Learning Congress 4 (second follow-up)) from the two rounds of survey.

### Analysis

Since teams in AB and BC had no opportunities for cross-connection, we only analyzed provincial networks. We developed two types of networks for this analysis: (1) any type of relationships among members belonging to different teams (knowing each other, communication, or collaboration) and (2) merged communication and collaboration networks. The latter would represent the networking activities among teams.

For each type of network, we calculated measures of network structure. The indicators of cohesion and connectivity included density (the proportion of possible ties that existed, overall and by region) and reciprocity (percent of mutual/bi-directional ties) [32]. E-I index (the ratio of the difference between the number of cross-region and within-region ties over total number of ties) [33] indicated the tendency to connect to other teams within and across regions. Network centralization (the inequality in distribution of incoming and outgoing ties) [34] indicated the potential hierarchy in the network. We also calculated indicators of network centrality to identify prominent and active QI teams.

We treated missing values (non-response) as zero, which assumes that there was no connection between teams. In order to assess whether teams in the same regions, and teams who belonged to the same organizational chains, were more likely to collaborate, we calculated quadratic assignment procedure (QAP) correlations of the connections among pairs of actors at each time point with being located in the same region and being affiliated to the same organizational chain [35].

We used the stochastic actor-oriented model (SAOM), performed in Rseina package in R, to assess the longitudinal dynamics of networks and to analyze how actor-level and network structural characteristics influenced the formation of new connections over the 6-month timeframe [36]. The SAOM model predicts the likelihood of creating or maintaining ties based on different network properties (e.g., density, reciprocity, and tendency of actors to connect to similar others) and actor attributes (e.g., geographical regions, and chain membership). The SAOM is one of the few models that permit the study of the evolution of networks and has been used in various fields [37–39]. The model assumes that the difference between networks at different time points is the result of many unobserved small changes occurring over the interval (also called micro-steps) through which network actors reconsider their relations with others (create, maintain (e.g., through reciprocation), or break) given their current conditions. The changes are assumed to be continuous Markov processes, meaning that the change at each micro-step is conditional on the previous timepoint [40]. Using an iterative algorithm, the SAOM simulates possible changes between time points to optimize the similarity between the simulated network and the observed network. After the model converges, the estimates of the effects in the model show the influence of personal, interpersonal, and overall network tendencies on creation or maintenance of network relationships over time. For example, if the actors have the tendency to reciprocate each other, the effect of reciprocity in creation of ties over time will be positive and statistically significant. Since we were interested in studying how SCOPE influenced the formation of new ties among QI teams, through co-participation in Learning Congresses and other collaborative activities, we used the *creation* function in SAOM models, predicting the formation of new network relations. The variables included in the SAOM model are explained in Table 1. To facilitate interpretation, we exponentiated the log odds coefficients to odds ratios.

**Table 1:** Definition of predictor variables in the SAOM models

Effects	Explanation
Rate function	On average how many opportunities each actor gets to make a change during each time period
Out-degree	Network density
Reciprocity	Actors’ tendency to make reciprocated ties
in-degree popularity	The tendency of actors with high in-degree to attract extra incoming ties because of their high in-degree (popular people become even more popular)
Out-degree activity	The tendency of actors with high out-degree to send extra outgoing ties because of their high out-degree (active people become even more active)
Region alter	The likelihood of actors in the region X to attract incoming ties compared with actors in the reference region
Region same	Whether the 2 actors in the same region tend to create new ties between each other
Chain same	Whether the 2 actors in the same chain tend to create new ties between each other

## Results

From 14 nursing homes that participated in SCOPE in Alberta, 10 at the first follow-up and 11 at the second follow-up provided their network information. Out of 17 nursing homes in BC, 11 at the first follow-up and 15 at the second follow-up provided their network information. In total, 13 teams in AB and 15 teams in BC provided information about their relationships before the SCOPE study at first or second follow-up assessments. All teams in Alberta were affiliated with organizational chains. Eleven teams in BC belonged to chains (Table 2).

**Table 2:** Characteristics of QI teams

	Alberta (AB)			British Columbia (BC)		
Region	AB Central and Calgary: 6 AB Edmonton: 8			Interior: 6 Fraser: 11		
Survey round	First round		Second round	First round		Second round
	Before	First follow-up	Second follow-up	Before	First follow-up	Second follow-up
Number of respondents	13	10	11	15	11	15

### Network visualization

**Fig. 1:**
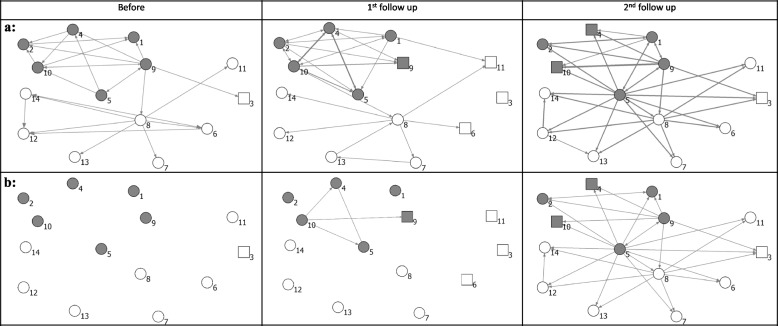
The relationships among teams in Alberta. **a** All types of relations. **b** Communication and collaboration only. Line thickness: tie strength, gray color: AB Central and Calgary, white color: AB Edmonton, circles: survey respondents

**Fig. 2:**
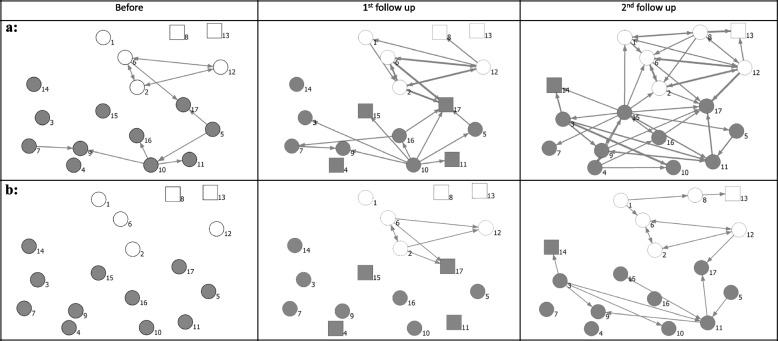
The relationships among teams in British Columbia. **a** All types of relations. **b** Communication and collaboration only. Line thickness: tie strength, gray color: BC-Fraser Health, white color: BC-Interior Health, circles: survey respondents

Figures 1A, B and 2A, B show the network of relations among teams before SCOPE and at first and second follow-ups. The networks in both provinces became denser over time. However, AB Calgary zone teams had been connected to each other relatively well prior to SCOPE and remained well-connected throughout the study. In AB, Central and Calgary zone teams mostly developed communication and collaboration relationships at the second follow-up.

The network in BC included many isolates prior to SCOPE. In contrast to the AB network that mostly included “communication and collaboration” relationships, the BC network included several new relations of teams only “knowing each other” at second follow-up.

### Network characteristics

Figures 1 and 2 also show that the total number of ties and overall density increased in both provinces over time. Table 3 provides descriptive statistics of social networks in three time points. In AB, cross-regional density increased from 2 to 10% at 2nd follow-up. The densities of connections within two regions fluctuated between different time points. The teams in AB Central and Calgary were connected relatively well prior to the SCOPE, and throughout the intervention, with a within-group density of 50%, 63%, and 53% prior to SCOPE and at 1st and 2nd follow-ups respectively. By contrast, in BC, density increased both within and across Fraser and Interior regions. Many teams in the Interior and Fraser regions did not have connections prior to SCOPE, and SCOPE facilitated the formation of connections both within and across regions.

**Table 3:** Descriptive statistics of networks in Alberta and British Columbia

	**All types of relations**						**Communication and Collaboration**					
	**Alberta**			**British Columbia**			**Alberta**			**British Columbia**		
**Measure**	**Before**	**1st follow-up**	**2nd follow-up**	**Before**	**1st follow-up**	**2nd follow-up**	**Before**^**a**^	**1st follow-up**	**2nd follow-up**	**Before**	**1st follow-up**	**2nd follow-up**
**Number of ties**	27	28	35	13	22	48	-	4	30	-	6	19
**Density overall**	15%	15%	19%	5%	8%	18%	-	2%	17%	-	2%	8%
**Density by region**	Edmonton: 18% Central and Calgary: 50% Between: 2%	Edmonton: 15% Central and Calgary: 63% Between: 2%	Edmonton: 16% Central and Calgary: 53% Between: 10%	Interior: 20% Fraser: 6% Between: 1%	Interior: 30% Fraser: 10% Between: 2%	Interior: 57% Fraser: 23% Between: 5%	-	Edmonton: 0 Central and Calgary: 13% Between: 0	Edmonton: 14% Central and Calgary: 40% Between: 10%	-	Interior: 13% Fraser: 0 Between: 2%	Interior: 30% Fraser: 8% Between: 1%
**Reciprocity**	29.6%	42.9%	22.9%	46%	27%	42%	-	0	13%	-	33.3%	42.1%
**In-degree centralization**	8.2%	15.4%	3.8%	7.7%	23.2%	26.1%	-	13.2%	6.6%	-	10.3%	11.8%
**Out-degree centralization**	3.9%	30.8%	80.8%	14.0%	35.7%	57.4%	-	20.9%	83.5%	-	16.5%	18.1%
**E-I index**	− 0.852	− 0.857	− 0.429	− 0.846	− 0.818	− 0.751	-	− 1	− 0.333	-	− 0.333	− 0.894

^a^We only asked about the existence of any types of relations prior to the SCOPE, so we did not calculate the frequency of communication or collaborations

The E-I index was negative for all networks, indicating a tendency to make connections within the region at all time points. The E-I index also increased in both provinces, especially in both regions of AB, indicating more connections were developed between regions after the intervention.

The increase in the overall density (all relations) in AB was predominantly attributed to communication and collaboration. The density of all relations at follow-up was 19%, and the density of communication and collaboration was 17%. This means that only about 2% of relations at second follow-up involved only knowing of each other. In contrast, all types of relations increased in BC. The density at 2nd follow-up was 18% for all relations, while only 8% for communication and collaboration, indicating a larger proportion of ties that involved knowing each other.

The reciprocity of communications and collaborations increased in both provinces, to 13% in Alberta, and to 42% in BC, indicating more bi-directional communications among teams. In-degree centralization of all relations showed an increasing trend in BC, which implies that the networks in BC became more centralized around a few popular actors. Out-degree centralization increased in both provinces, which implies that already active teams in both provinces became even more active.

Table 4 shows the QAP correlation of having any type of network connection (knowing, communication, or collaboration) with co-locating in the same region and belonging to the same organizational chain for pairs of actors at each time point. The correlations between chain relationships and networks in AB and BC prior to SCOPE and at first follow-up are statistically significant and positive, meaning that teams belonging to the same chain were more likely to be connected before the SCOPE intervention. The correlation between chain co-membership and network ties decreased at follow-up in both provinces, indicating that the teams probably developed more relations to others outside of their organizational chains. The correlation between being in the same region and networks were statistically significant at all time points.

**Table 4:** The QAP correlation (*p* value) of network relations with being in the same region and co-affiliating to the same chain

	Co-affiliation to the same chain	Being in the same region
**Alberta**		
Before	0.22 (0.02)	0.396(0.001)
1st follow-up	0.29 (0.009)	0.444(0.001)
2nd follow-up	0.14 (0.06)	0.232(0.004)
**British Columbia**		
Before	0.37 (0.001)	0.183(0.014)
1st follow-up	0.40 (0.001)	0.354(0.001)
2nd follow-up	0.23 (0.005)	0.301(0.001)

### Statistical modeling

Table 5 shows the results of SAOM for all-relation networks (knowing each other, communication, and collaboration) in AB and BC. Each parameter in the SAOM had a convergence *t*-ratio of less than 0.1, and the overall *t*-ratios for the two models were less than 0.15, indicating a satisfactory convergence. The model estimates in Table 5 illustrate the effects of various actor-level, dyadic, and structural factors on creating new ties between teams.

**Table 5:** SAOM models of network evolution in Alberta and British Columbia

	**Alberta**	**British Columbia**
	**OR (95% CI)**	**OR (95% CI)**
**Rate effects**		
Rate parameter period 1	6.23 (1.72,10.75)*	0.98 (0.37,1.58)*
Rate parameter period 2	28.67 (− 6.64,63.98)	7.47 (3.50,11.44)*
**Network factors**		
Out-degree (density)	0.02 (0.001, 0.63)	1.19 (0.02, 92.62)
Reciprocity	1.11 (0.54, 2.29)	1.41 (0.67, 2.96)
In-degree—popularity (sqrt)	0.182 (0.005, 6.52)	0.72 (0.18, 2.85)
Out-degree—activity (sqrt)	4.12 (1.24, 13.66)*	0.50 (0.02, 13.58)
Region	Central and Calgary: 9.84 (1.38, 60.46)*	Fraser: 0.44 (0.16, 1.21)
Being in the same region	10.07 (2.42, 41.93)*	12.75 (4.06, 12.75)*
Affiliating to the same chain	3.29 (0.78, 13.88)	2.30 (0.48, 10.93)

^*^p < 0.05

The rate parameters (an indicator of overall connectivity) show that in both provinces the network connectivity increased at 1st follow-up compared to before SCOPE. The increase in connectivity at 2nd follow-up (compared to 1st follow-up) was statistically significant in BC. Reciprocity and in-degree popularity showed non-significant effects in AB and BC, indicating that there was no significant change in networks towards becoming more reciprocal or more centralized from first to second follow-ups. In AB, out-degree-activity exerted a significant positive effect on tie creation, meaning that already active teams were more likely to become even more active at follow-up, as reflected in the 4.1 times odds ratio of creating new ties. The odds ratio of creating new ties in AB Central and Calgary (versus AB Edmonton) was 9.8, which indicates that teams in the Central and Calgary zone were more likely to make new connections. In both AB and BC, teams in the same regions had higher odds of building new ties with each other, in comparison with teams that were not in the same region (odds ratio of 10 in AB and 12.8 in BC). In addition, belonging to the same organizational chains had a positive, though non-significant, effect on forming new ties.

## Discussion

We used longitudinal network analysis to assess the effect of participating in the SCOPE study on networks of care aide-led QI teams located in two Canadian provinces. While increasing connectivity was not the focus of SCOPE, we found that the overall network connectivity within and across regions increased over time in both provinces, which included connections to new teams as well as strengthening existing relations (from knowing each other to communication and collaboration). The gradual increase of connectivity over time suggests that SCOPE was successful in building relationships between teams across nursing homes. This could be attributed to the participatory nature of the SCOPE intervention that provided various opportunities to collaborate, including Learning Congresses where teams were encouraged to interact and share knowledge and experiences, and regular progress meetings with a quality improvement advisor that spanned across teams. SCOPE was also subject to a rigorous process evaluation where teams gathered to discuss their experiences of participation and progress with their QI intervention [41]. Early evidence from the SCOPE intervention showed that its team-based, collaborative structure facilitated the perception of communication and support among the members of QI teams within nursing homes, as reflected in cohesion scores by team members [42, 43].

In our analysis, we found that geographic proximity and co-membership in organizational chains were important predictors of connectivity before, and during SCOPE. In both provinces, teams were more likely to connect to others in the same region and those who belonged to the same organizational chains. This may explain higher network density before SCOPE in AB (compared to BC), since 10 out of 14 AB teams shared chain memberships and most teams in AB- Central and Calgary were geographically close to each other, as opposed to BC, in which 8 out of 17 shared chains, and many BC teams, particularly in the Fraser Health region, were geographically dispersed, independent, family-run nursing homes. Proximity is an important factor in the creation of ties between teams [44–46]. Geographic proximity facilitates knowledge diffusion and information exchange through serendipitous communications between neighboring actors [47, 48]. Organizational proximity, on the other hand, occurs when network actors share institutional membership, such as belonging to chains. Our findings suggest that nursing homes that were geographically closer were more likely to build new relations. Co-affiliation to chains (which potentially involved shared standards of care, leadership, staffing, and prior recognition of each other) was significantly associated with relationships at each timepoint (but not a significant predictor of building new ties), indicating stable connectivity among nursing homes belonging to same chains throughout the study. Evidence from other contexts also indicates that healthcare organizations with shared governance, such as nursing homes belonging to the same chains, usually have better opportunities to form connections and access common resources or may share staff and knowledge, which can facilitate partnership and influence the quality of care [49, 50].

In BC, many teams did not have connections before SCOPE (as reflected in the smaller density) but developed a dense and highly reciprocated network over time, which was reflected in the increased knowledge of other teams and development of communication and collaboration ties. The larger increase in reciprocity in BC compared to AB implies that independent teams probably benefited more from the network-altering nature of SCOPE and used this opportunity to build connections with new partners. In other contexts, healthcare professionals, particularly in remote and rural areas, have expressed needs to belong to communities of support and learning, to help addressing isolation, and finding others with similar challenges and experiences [51].

A common pattern in both provinces was an increasing trend of out-degree centralization. Based on the SAOM model results, teams in AB that were already active became even more active after the intervention. This implies that the SCOPE intervention provided opportunities that were disproportionately utilized by teams who were already outgoing and active; thus, less-outgoing teams benefited less from the networking aspects of the intervention. This suggests that, if identified and engaged, active network members might be intentionally engaged to transmit new ideas, and help developing trust and sustainable relationships, and may form the next generation of opinion leaders in the network [52].

This study has several limitations, including the lack of parallel control group, and inability to assess the network building impact of various SCOPE components. In addition, we did not assess the relationship between connectivity and network positions with QI outcomes and satisfaction with the intervention. The respondents to the surveys were the team sponsors (most often a unit manager) and senior sponsors (most often a director of care from the facility), and not every team participated in both rounds. This may limit the perspectives reflected in the social networks, even though they were members of the SCOPE QI teams, along with the care aides, and participated in SCOPE sessions and were actively involved in reporting the progress.

Larger-scale studies can assess and optimize the impact of different strategies included in complex team-based interventions using advanced experimental designs, such as factorial design, and using strategies for intervention optimization, such as multiphase optimization strategy (MOST) [53]. The findings should be interpreted in light of the nature and dynamics of collaboration networks among nursing homes in study provinces and might have limited generalizability to other contexts.

## Conclusions

Our findings provide empirical evidence for the formation of information-sharing and collaboration networks among QI teams across nursing homes participating in the SCOPE intervention. We assume that the participatory, facilitated, and collaborative nature of SCOPE helped teams build and strengthen relations, which were more prominent in proximate (geographically and organizationally) nursing homes. Teams that were already outgoing prior to SCOPE became more connected. On the other hand, teams that did not have prior opportunities to connect appeared to use SCOPE to build new ties.

These findings may inform the development of more intentional network-altering interventions to leverage existing relationships and new networking opportunities to facilitate and sustain QI and implementation programs. We summarize the implications of these findings for network-altering interventions among nursing home teams:

- Team activities through participatory events (such as Learning Congresses) are effective in shaping communication and collaboration ties across nursing home teams [8]. When formed, many of these relations are sustainable. Opportunities should be provided for joint activities and peer feedback across teams.
- Participants in training programs do not benefit equally from networking opportunities. Already well-connected teams are more likely to make more connections over time (known as “rich get richer” phenomenon) [54]. Special attention should be given to empowering peripheral and isolated teams.
- Geographical proximity and chain co-affiliation are important predictors of connectivity. This demonstrates the influence of homophily (being connected to others with similar features) as an important tendency in social networks [55]. Active interventions are needed to develop networks that bridge across geographical and organizational silos.

SCOPE was not designed to be a network-altering intervention; however, it clearly does influence and re-shape social networks among participating teams. While generally unacknowledged, the success of complex team-based interventions such as SCOPE is enhanced through development of strategies that intentionally leverage and alter networks to facilitate “enriched” information exchange and access to support and resources [6].

### Supplementary Information

**Supplementary Materials 1. **

**Supplementary Materials 2. **

## Data Availability

The datasets during and/or analyzed during the current study available from the corresponding author on reasonable request.
