# Supplementary material for: Building a communication and support network among quality improvement teams in nursing homes: a longitudinal study of the SCOPE trial
Source: Implement Sci Commun. 2024 Mar 4;5:19. doi: 10.1186/s43058-024-00559-y (PMC10913450; doi:10.1186/s43058-024-00559-y)
Supplement: Supplementary file 2 — Supplementary Materials 2. [file 43058_2024_559_MOESM2_ESM.docx]

SCOPE networking survey

Friday, August 31, 2018

This survey is about how SCOPE quality improvement teams communicate with other SCOPE teams.

We ask that you complete this survey within 2 weeks of receiving the link to it.

**What are the possible benefits of taking part in the study?**

Your answers to the survey will help us understand how we can work with teams to help them connect and support one another in quality improvement.

**What are the potential risks of taking part in the study?**

There are no known risks to participating in this study.

**How will we protect your confidentiality?**

Your name and your team’s name will not be shared and will not appear in any reports and publications.

**Do I have to take part in the study?**

Participation in the study is voluntary and you can stop at any time up to one month after the fifth and last round of the survey (this is approximately 8 months after the last SCOPE Learning Congress). If you want your responses to be removed, please contact the researchers listed below.

**What will we do with the data?**

-We will analyze the data that we collect from you to “map” communications between teams.

-Your identity and that of your team will not be revealed to anyone outside the study. All of the analysis will be done at the team level (not individual level).

-All data will be stored and analyzed through a secure system hosted by the University of Alberta (https://www.ualberta.ca/nursing/research/supports-and-services/hrdr).

-TREC’s data practices are consistent with the Canadian Institutes of Health Research (CIHR) Best Practices for Protecting Privacy in Health Research and the Tri Council Policy Statement on Ethical Conduct for Research Involving Humans (TCPS2). All data will be securely retained in the system at least until 2027.

**Ethics review:**

The plan for this study has been reviewed by a Research Ethics Board at the University of Alberta. If you have questions about your rights or how research should be conducted, you can call (780) 492-2615.  This office is independent of the researchers. The study ethics ID is Pro00082213.

Thank you for considering participation in this study.

If you have any questions and concerns regarding this survey, please contact the researchers or SCOPE administrative staff through the following contact information:

Reza Yousefi Nooraie (researcher)

Phone: 905-616-7554

Email: r.yousefinooraie@utoronto.ca

Judith Palfreyman (SCOPE Trial coordinator)

Phone: 780-492-8473

Email: judith.palfreyman@ualberta.ca

### Respondent’s name

### How familiar are you with the communications and collaborations that occur between your SCOPE quality improvement team(s) and SCOPE teams in other facilities.

○ 1: Little or No Familiarity

○ 2: Somewhat Familiar

○ 3: Very Familiar

Would you please write the name and contact (email and/or phone number) of the person in your facility who is familiar with the communications and collaborations that occur between the quality improvement team in your facility and other SCOPE teams?

### Region

○ Alberta North

○ Alberta South

○ Fraser Health

○ Interior Health

### Facility name

○

### Please check the item that best describes your SCOPE team's current relationship with other SCOPE teams located in your region.

### Please answer on behalf of your team.

### We are interested in relationships between anyone involved in the SCOPE team (such as sponsors, QI team leads, or other levels).

|  | No relationships To date we have developed little or no relationships with this team | Knowing each other We have made connections with members of this team about who they are, what they are working on, and how we can help one another | Communication We share useful information and ideas with the members of this team when opportunities arise | Collaboration We informally or formally work together with this team to achieve common goals |
| --- | --- | --- | --- | --- |
| Team 1 | ○ | ○ | ○ | ○ |
| Team 2 |  |  |  |  |

Do you want to add any other SCOPE teams from facilities located in other regions?

………

### Before the SCOPE study began, did anyone from your facility communicate/interact with anyone in any of the other SCOPE facilities located in your region?

### Please answer to the best of your knowledge on behalf of your facility.

|  | No relationships We had little or no relationships with people working at this facility. | Communication or collaboration We shared useful information and ideas or worked together informally or formally. | I do not know |
| --- | --- | --- | --- |
| Team 1 | ○ | ○ | ○ |
| Team 2 |  |  |  |

Do you want to add any other SCOPE teams from facilities located in other regions?

………
